# Integrating microRNA and mRNA expression profiles of acute promyelocytic leukemia cells to explore the occurrence mechanisms of differentiation syndrome

**DOI:** 10.18632/oncotarget.11989

**Published:** 2016-09-13

**Authors:** Yingmei Zhang, Jinxiao Hou, Fei Ge, Fenglin Cao, Haitao Li, Ping Wang, Mengyuan Xu, Peng Song, Xiaoxia Li, Shuye Wang, Jinmei Li, Xueying Han, Yanhong Zhao, Yanhua Su, Yinghua Li, Shengjin Fan, Limin Li, Jin Zhou

**Affiliations:** ^1^ Central Laboratory, The First Affiliated Hospital, Harbin Medical University, Harbin, China; ^2^ Department of Hematology, The First Affiliated Hospital, Harbin Medical University, Harbin, China; ^3^ Department of Neonatology, The First Affiliated Hospital, Harbin Medical University, Harbin, China

**Keywords:** acute promyelocytic leukemia, differentiation syndrome, microRNA, mRNA, microarrays

## Abstract

The pathogenesis of therapy-induced differentiation syndrome (DS) in patients with acute promyelocytic leukemia (APL) remains unclear. In this study, mRNA and microRNA (miRNA) expression profiling of peripheral blood APL cells from patients complicated with vs. without DS were integratively analyzed to explore the mechanisms underlying arsenic trioxide treatment-associated DS. By integrating the differentially expressed data with the data of differentially expressed microRNAs and their computationally predicted target genes, as well as the data of transcription factors and differentially expressed target microRNAs obtained from a literature search, a DS-related genetic regulatory network was constructed. Then using an EAGLE algorithm in clusterViz, the network was subdivided into 10 modules. Using the Kyoto Encyclopedia of Genes and Genomes (KEGG) database the modules were annotated functionally, and three functionally active modules were recognized. The further in-depth analyses on the annotated functions of the three modules and the expression and roles of the related genes revealed that proliferation, differentiation, apoptosis and infiltration capability of APL cells might play important roles in the DS pathogenesis. The results could improve our understanding of DS pathogenesis from a more overall perspective, and could provide new clues for future research.

## INTRODUCTION

The advent of differentiation therapy has transformed acute promyelocytic leukemia (APL) from a disease with a poor prognosis to the most frequently curable acute leukemia. However, differentiation syndrome (DS), a serious and sometimes life-threatening complication of differentiation therapy, is observed in approximately 25% of APL patients [1–3] and approximately 15% of deaths during APL induction therapy are related to DS [2, 4, 5]. DS has become the second- or third-leading cause of death during induction therapy for APL. Moreover, the occurrence of DS not only forebodes an increasing risk of death but also indicates a higher recurrence risk [6, 7].

The molecular mechanisms underlying the pathogenesis of DS remain unknown. The differentiating APL cells should play a crucial role in the development of DS since DS is not observed during consolidation or maintenance therapy with all-trans-retinoic acid (ATRA) or/and arsenic trioxide (ATO) in APL patients or during ATRA or ATO treatment in non-APL malignancies [8, 9].

In this study, mRNA and microRNA (miRNA) expression profiling of APL cells from fresh peripheral blood were integratively analyzed to explore the mechanisms underlying ATO treatment-associated DS.

## RESULTS

### Clinical features were comparable between DS and non-DS groups

The clinical features were comparable between the two groups, except the baseline white blood cell (WBC) count, and the WBC count at the time of blood sampling which are both risk factors for developing DS [1, 10] (Table S1).

### Differentially expressed genetic profile and differentially expressed miRNA profile

A total of 1701 differentially expressed genes were detected between the DS and non-DS groups, including 741 up-regulated genes and 960 down-regulated genes in the DS group. A total of 45 differentially expressed miRNAs were detected, including 18 up-regulated miRNAs and 27 down-regulated miRNAs in the DS group (Table 1).

**Table 1:** List of differentially expressed miRNAs between the patients with and without differentiation syndrome

	Upregulated miRNA	*P* value	Fold change		Downregulated miRNA	*P* value	Fold change
1	hsa-miR-548g	0.003	5.39	1	hsa-miR-30d	<0.001	0.19
2	hsa-miR-720	0.004	2.99	2	hsa-miR-625*	0.006	0.14
3	hsa-miR-17	0.005	5.07	3	hsa-miR-217	0.011	0.19
4	solexa-555-1991	0.006	5.11	4	hsa-miR-181d	0.012	0.19
5	hsa-miR-10b*	0.008	5.353	5	hsa-miR-152	0.015	0.29
6	hsa-miR-1274a	0.011	2.17	6	hsa-miR-191	0.016	0.37
7	hsa-miR-137	0.012	6.24	7	hsa-miR-15b	0.019	0.28
8	hsa-miR-93	0.012	4.63	8	hsa-miR-600	0.021	0.29
9	hsa-miR-106a	0.014	6.61	9	hsa-miR-425	0.021	0.43
10	hsa-miR-18a	0.017	5.68	10	hsa-miR-421	0.021	0.17
11	hsa-miR-1274b	0.023	4.56	11	hsa-miR-454*	0.023	0.28
12	hsa-miR-132*	0.025	3.84	12	hsa-miR-1285	0.025	0.15
13	hsa-miR-133a	0.027	4.05	13	hsa-miR-30c	0.025	0.23
14	hsa-miR-204	0.029	4.96	14	hsa-miR-202*	0.027	0.46
15	hsa-miR-1280	0.030	3.67	15	hsa-miR-150	0.031	0.42
16	hsa-miR-92a	0.031	2.24	16	hsa-miR-548b-3p	0.033	0.49
17	hsa-miR-101*	0.032	7.75	17	hsa-miR-10a	0.038	0.24
18	hsa-miR-16-1*	0.032	2.52	18	hsa-miR-345:9.1	0.044	0.37
19	hsa-let-7i*	0.038	5.13				
20	hsa-miR-29a*	0.039	5.91				
21	hsa-miR-148b*	0.039	3.75				
22	hsa-miR-661	0.040	3.93				
23	hsa-miR-27b*	0.043	2.61				
24	hsa-miR-431	0.046	2.08				
25	hsa-miR-210	0.047	2.52				
26	hsa-miR-302c*	0.048	3.05				
27	hsa-miR-221	0.049	2.67				

### Profiles of the differentially expressed miRNAs/differentially expressed target genes regulatory pairs and differentially expressed transcription factors/differentially expressed miRNAs regulatory pairs

We identified 196 pairs of differentially expressed miRNAs/differentially expressed target genes, involving 24 differentially expressed miRNAs and 138 differentially expressed genes.

We identified 12 pairs of differentially expressed transcription factors/differentially expressed miRNAs, involving four differentially expressed transcription factors and eight differentially expressed miRNAs (Table 2). The majority of the detected transcription factors showed a regulatory effect on the corresponding miRNA consistent with the previous reports, but three transcription factors showed an opposite regulatory effect. In fact, there are multiple factors that could regulate miRNA expression. Perhaps some unknown regulating factors existed simultaneously, or maybe under different conditions, the regulatory effect on miRNA by transcription factor is naturally different (promotive or inhibitive), such as the regulation of miR-17, miR-106a, and miR-93 expression by E2F1.

**Table 2:** Regulatory effects of transcription factors on miRNA expression: comparison between this study and previous reports

Transcription factor	miRNA	Regulatory effect		ID of the reference paper in PubMed
		This study	Previous report	
PPP3R1	miR-133a	Activation *	Inhibition	20177001
E2F1	miR-106a	Activation	Regulation	19034270
E2F1	miR-15b	Inhibition *	Activation	20404092; 21454377
E2F1	miR-17	Activation	Regulation	19034270; 19066217
E2F1	miR-18a	Activation	Activation	19066217
E2F1	miR-93	Activation	Regulation	19034270
MYC	miR-106a	Activation	Regulation	17943719; 20878079
MYC	miR-17	Activation	Regulation	20008931; 20643754; 20878079
MYC	miR-18a	Activation	Activation	17943719; 19066217
MYC	miR-221	Activation	Activation	17943719
MYC	miR-93	Activation *	Inhibition	20878079
VHL	miR-210	Activation	Activation	18316553

* The regulatory effect observed in this study is opposite to that in the literature.

### DS-related gene-regulatory network was constructed and subdivided into 10 modules

**Figure 1:**
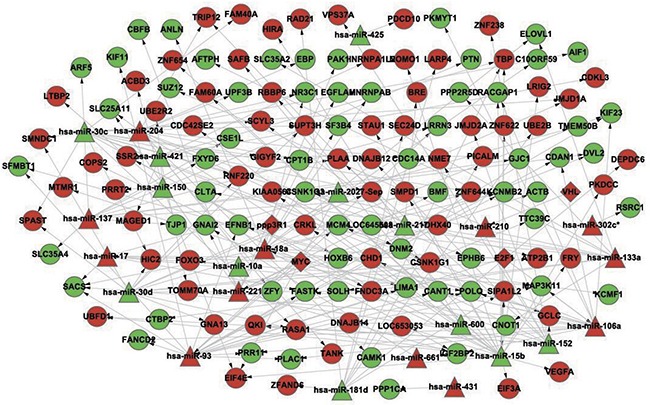
The constructed gene-regulatory network linked to differentiation syndrome In the figure, diamond nodes represent differentially expressed transcription factors, triangle nodes represent differentially expressed miRNAs, and round nodes represent differentially expressed miRNA target genes. Red nodes and green nodes indicate that the gene or miRNA is upregulated and downregulated, respectively, in the patients with differentiation syndrome compared with patients without differentiation syndrome. A directed edge indicates that a node (transcription factor or miRNA) can regulate the expression of another node (miRNA or gene).

Based on the identified pairs of differentially expressed miRNAs/differentially expressed target genes and differentially expressed transcription factors/differentially expressed miRNAs, a DS-related gene-regulatory network was constructed, which consisted of 165 nodes and 206 edges (Figure 1).

**Figure 2:**
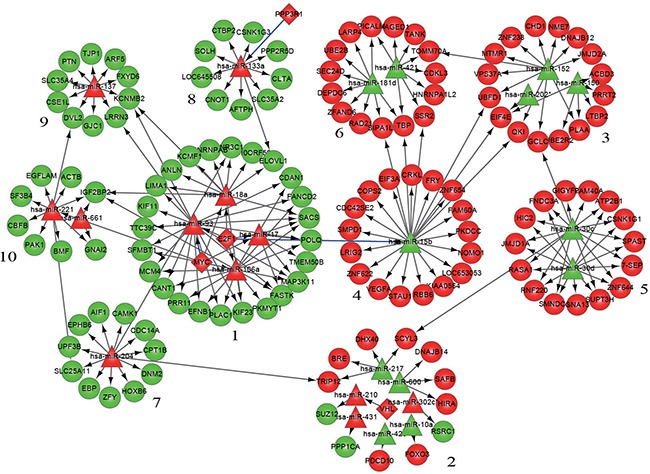
Modularization of the gene-regulatory network linked to differentiation syndrome In the figure, the numbers are the serial numbers of modules. Diamond nodes represent differentially expressed transcription factors, triangle nodes represent differentially expressed miRNAs, and round nodes represent differentially expressed miRNA target genes. Red nodes and green nodes indicate that the gene or miRNA is upregulated and downregulated, respectively, in the patients with differentiation syndrome compared with patients without differentiation syndrome. A directed edge indicates that a node (transcription factor or miRNA) can regulate the expression of another node (miRNA or gene). A blue edge indicates that the regulation of miRNA expression by a transcription factor in this study is opposite to that reported in the literature.

The entire network was then subdivided into 10 modules; each module had at its center one or more miRNAs with or without being accompanied by 1-2 transcription factors and surrounded by 8-24 genes whose expression could be regulated by the miRNA in the center. The details of miRNAs, genetic composition, and regulatory relationships in the 10 modules are shown in Figure 2 and Table 3.

**Table 3:** Distribution of nodes and edges in each module

	Module										Intermodule
	1	2	3	4	5	6	7	8	9	10	
Number of nodes	30	20	19	18	17	17	12	11	11	10	0
Number of edges	52	13	18	17	27	17	11	10	10	10	21

### Results derived from functional annotations of each module

The Kyoto Encyclopedia of Genes and Genomes (KEGG) pathway enrichment analysis on each modular gene set demonstrated that the genes in modules 1, 4, and 10 were enriched in nine, three, and six KEGG pathways, respectively; the genes of modules 2, 3, and 6 were each enriched in one KEGG pathway; and the genes in the remaining modules did not show significant enrichment in any KEGG pathway (Table 4). In addition to the enrichment in multiple KEGG pathways, the genes in modules 1, 4, and 10 had a relatively greater number of links with the surrounding modules than did the other modules (Figure 2), as evidenced by the fact that only two links among a total of 21 intermodular links were unrelated to modules 1, 4, and 10.

**Table 4:** Functional annotation of modules by KEGG database

No.	Module	Enriched KEGG pathway	Genes
1	1	Cell cycle (Figure 3A)	*MCM4, PKMYT1, MYC, E2F1*
2	1	Pathways in cancer (Figure 3B)	*E2F1, MYC*
3	1	p53 signaling pathway (Figure 3C)	*E2F1, MYC*
4	1	MAPK signaling pathway (Figure 3D)	*MYC, MAP3K11*
5	1	Chronic myeloid leukemia	*E2F1, MYC*
6	1	Small cell lung cancer	*E2F1, MYC*
7	1	Bladder cancer	*E2F1, MYC*
8	1	HTLV-I infection	*E2F1, MYC*
9	1	Hepatitis B	*E2F1, MYC*
10	2	Ubiquitin mediated proteolysis	*TRIP12, VHL*
11	3	Protein processing in endoplasmic reticulum	*DNAJB12, PLAA*
12	4	Focal adhesion (Figure 4A)	*CRKL, VEGFA*
13	4	Pathways in cancer (Figure 4B)	*CRKL, VEGFA*
14	4	Renal cell carcinoma	*CRKL, VEGFA*
15	6	Protein processing in endoplasmic reticulum	*SEC24D, SSR2*
16	10	Focal adhesion (Figure 5A)	*PAK1, ACTB*
17	10	Regulation of actin cytoskeleton (Figure 5B)	*PAK1, ACTB*
18	10	Chemokine signaling pathway (Figure 5C)	*PAK1, GNAI2*
19	10	Leukocyte transendothelial migration (Figure S3D)	*ACTB, GNAI2*
20	10	Axon guidance	*PAK1, GNAI2*
21	10	Proteoglycans in cancer (Figure 5E)	*PAK1, ACTB*

### Validation of differentially expressed mRNAs and miRNAs by real-time quantitative reverse transcription polymerase chain reaction (qRT-PCR)

All the 24 miRNAs included in the 10 modules and 7 mRNAs in modules 1, 4, and 10 were selected for validation (Table 5). There were similar up- and down-regulation profiles between microarray and real-time qRT-PCR in up to 86% of the mRNA (all except ACTB) and 75% of the miRNA (all except miR-93, miR-661, miR-202*, miR-302c*, miR-425 and miR-431). All of the mRNAs and miRNAs except *ACTB*, *CRKL*, miR-93, miR-661, miR-133a, miR-150, miR-204, miR-302c* and miR-425 were found to be significantly differentially expressed (P<0.05) between non-DS and DS groups.

**Table 5:** Expression levels of interesting miRNAs/mRNAs in Non-DS group and DS group determined by real-time qRT-PCR (mean ± standard deviation)

No.	miRNA/mRNA	Non-DS group	DS group	*P* value
1	miR-15b	1 ± 0.48	0.46 ± 0.08	0.022
2	miR-17	1 ± 0.53	3.03 ± 1.58	0.014
3	miR-18a	1 ± 0.42	4.01 ± 1.97	0.004
4	miR-93	1 ± 0.28	0.89 ± 0.34	0.554
5	miR-106a	1 ± 0.34	2.58 ± 1.24	0.013
6	miR-221	1 ± 0.39	1.83 ± 0.68	0.027
7	miR-661	1 ± 0.38	0.68 ± 0.29	0.135
8	miR-10a	1 ± 0.54	0.31 ± 0.11	0.012
9	miR-133a	1 ± 0.51	1.72 ± 0.84	0.101
10	miR-137	1 ± 0.66	3.81 ± 2.14	0.012
11	miR-150	1 ± 0.43	0.72 ± 0.31	0.231
12	miR-152	1 ± 0.61	0.22 ± 0.08	0.011
13	miR-181d	1 ± 0.67	0.15 ± 0.11	0.012
14	miR-202*	1 ± 0.28	1.46 ± 0.31	0.022
15	miR-204	1 ± 0.6	1.75 ± 0.93	0.127
16	miR-210	1 ± 0.46	2.82 ± 1.57	0.021
17	miR-217	1 ± 0.52	0.29 ± 0.24	0.013
18	miR-302c*	1 ± 0.44	0.81 ± 0.27	0.389
19	miR-30c	1 ± 0.47	0.47 ± 0.23	0.032
20	miR-30d	1 ± 0.28	0.61 ± 0.12	0.010
21	miR-421	1 ± 0.64	0.31 ± 0.14	0.027
22	miR-425	1 ± 0.58	1.88 ± 1.06	0.104
23	miR-431	1 ± 0.33	0.66 ± 0.08	0.034
24	miR-600	1 ± 0.69	0.24 ± 0.11	0.024
25	*E2F1*	1 ± 0.53	2.08 ± 0.94	0.035
26	*c-MYC*	1 ± 0.62	2.03 ± 0.72	0.024
27	*VEGF-A*	1 ± 0.46	2.13 ± 1.08	0.040
28	*CRKL*	1 ± 0.28	1.36 ± 0.36	0.079
29	*ACTB*	1 ± 0.3	1.15 ± 0.19	0.313
30	*PAK1*	1 ± 0.42	0.32 ± 0.12	0.004
31	*GNAI2*	1 ± 0.33	0.65 ± 0.08	0.031

## DISCUSSION

In this study, we developed an integrative analysis method for miRNA and mRNA expression profiles. First, the disease-related gene-regulatory network was constructed in turn of 3 layers of biomolecules, i.e. differentially expressed transcription factors, differentially expressed target miRNAs, and differentially expressed target genes. Second, based on the characteristics of modularity and hierarchy in the topological structure of biological networks [11, 12], the constructed gene-regulatory network were modularized. Each module was structurally independent, i.e. the intramodular nodes were tightly connected with each other, while the links between the nodes in different modules were sparse. To a certain extent, a biological network module can represent a functional unit of a biological process; biomolecules in same module often show much greater similarity in function than those among different modules [11, 12]. Therefore, instead of a complete description of complex network structures, modularization of biological networks can simplify analysis processes and further our understanding of the mechanisms underlying biological functions. There are several module recognizing methods to choose from for each type of biological network. In this study a clustering method was chose. Third, each module was annotated functionally by KEGG pathway enrichment analysis of the proteins encoded by the intramodular genes. Enrichment analysis of miRNA targets is a standard technique to elucidate regulatory functions of miRNAs in gene regulatory networks [13].

The enrichment analysis revealed that the proteins encoded by the genes in modules 1, 4, and 10 were significantly enriched in multiple KEGG pathways (Table 4). Hence, the functions of the genes in the three modules were further analyzed by mapping corresponding encoding proteins to the enriched KEGG pathways.

**Figure 3:**
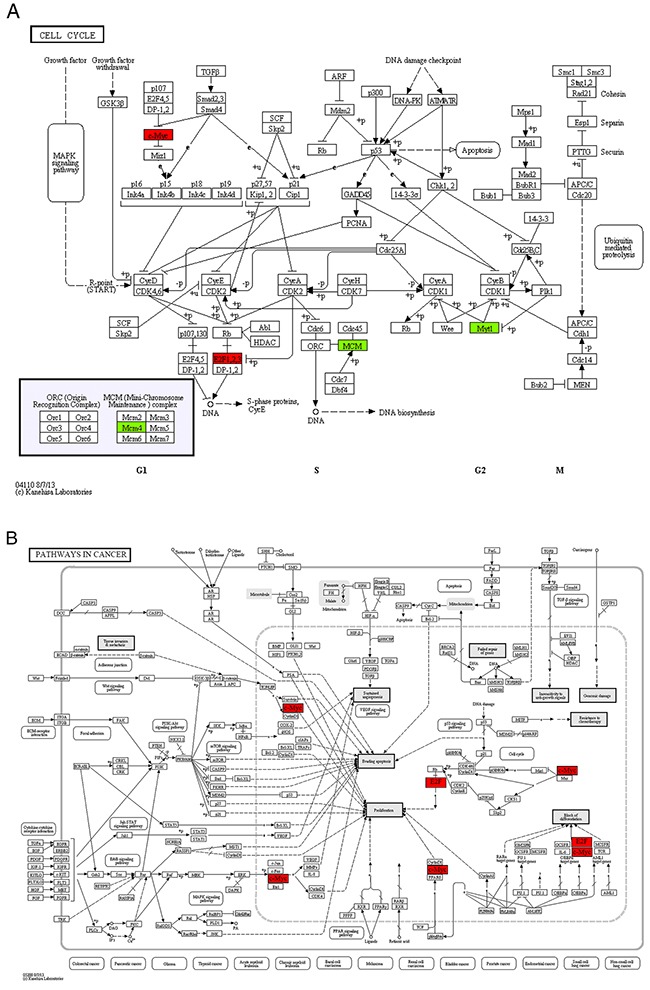
The maps of KEGG pathways that are enriched for genes in module 1 In each map, the red or green boxes show the localization of genes of module 1 in the KEGG pathway. Red and green indicate that the gene is upregulated and downregulated, respectively, in the patients complicated with differentiation syndrome. In each map, the red or green boxes show the localization of genes of module 1 in the KEGG pathway. Red and green indicate that the gene is upregulated and downregulated, respectively, in the patients complicated with differentiation syndrome.

In module 1, four of the nine enriched KEGG pathways, cell cycle, pathways in cancer, p53 signaling pathway and MAPK signaling pathway, were non-disease-specific (Table 4). The location and role of the genes of module 1 in the 4 KEGG pathways were analyzed (Figure 3) and it was found that these genes played an important role in cell proliferation, differentiation, and apoptosis. The two transcription factors, E2F1 and MYC, were functionally active and involved in all 9 enriched KEGG pathways. E2F1 is a member of the E2F family of transcription factors. The E2F family plays a crucial role in the control of cell cycle. As an important cell cycle regulatory factor, E2F1 can promote cell cycle transition from G0/G1 phase to S phase via the E2F/RB pathway, accordingly accelerate cell proliferation (Figure 3A, 3B). E2F1 can also mediate p53-dependent/independent apoptosis (Figure 3C). MYC, i.e. C-MYC, is a multifunctional transcription factor. C-MYC can drive cell proliferation by up-regulating cyclins and down-regulating p21 (a cyclin-dependent kinase inhibitor) (Figure 3A–3C), promote cell apoptosis through down-regulating Bcl-2 expression, and inhibit cell differentiation (Figure 3B, 3C). E2F1 and c-MYC could also exert their functions through increasing the expression of miRNAs, such as miR-17, miR-18a and miR-106a. In the DS group, the expression levels of both *E2F1*, *c-MYC* mRNAs and miR-17, miR-18a, miR-106a were up-regulated, indicating that in patients with DS, the proliferation and apoptosis of APL cells in peripheral blood were enhanced and the differentiation was attenuated.

**Figure 4:**
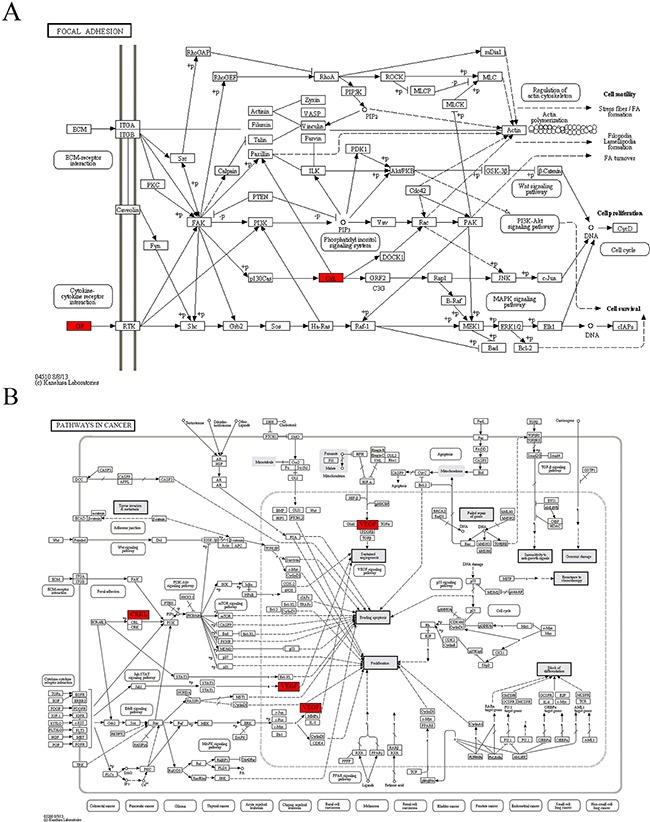
The maps of KEGG pathways that are enriched for genes of module 4 In each map, the red boxes show the localization of genes of module 4 in the KEGG pathway. Red indicates that the gene is upregulated in the patients complicated with differentiation syndrome.

In module 4, vascular endothelial growth factor-A (VEGF-A) and CRKL were highly functionally active and involved in both “focal adhesion” and “pathways in cancer” pathways annotated in module 4 (Table 4, Figure 4). As a secretory protein, VEGF-A can specifically act on endothelial cells and induce angiogenesis, as well as enhance the permeability of blood vessels. CRKL can regulate the reassembly of the cellular actin cytoskeleton and cellular migration and is an indispensable protein required for local cellular adhesion. Both *VEGF-A* and *CRK*L are target genes of miR-15b. The real-time qRT-PCR analysis revealed that the expression of *CRKL* mRNAs was up-regulated in the DS group, but the difference was not statistically significant. The expression of *VEGF-A* mRNAs was significantly up-regulated while miR-15b was significantly down-regulated in the DS group, indicating that in patients with DS, the invasion and infiltration capacity of APL cells in peripheral blood was enhanced.

**Figure 5:**
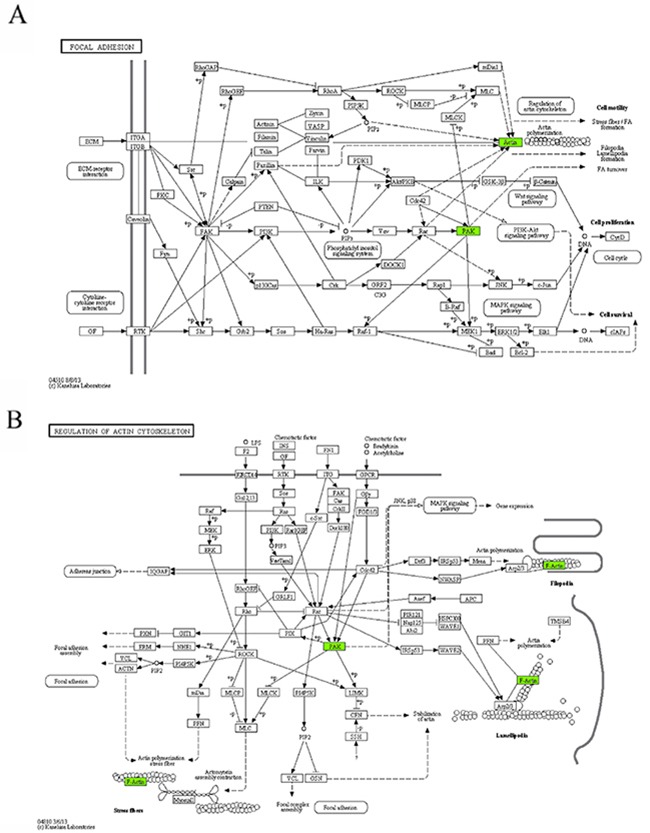
The maps of KEGG pathways that are enriched for genes of module 10 In each map, the green boxes show the localization of genes of module 10 in the KEGG pathway. Green indicates that the gene is downregulated in the patients complicated with differentiation syndrome. In each map, the green boxes show the localization of genes of module 10 in the KEGG pathway. Green indicates that the gene is downregulated in the patients complicated with differentiation syndrome.

Six KEGG pathways annotated in module 10 were closely associated with cell invasion and infiltration capacity (i.e., cellular adhesive ability, motor ability, and responsiveness to chemokines). The genes in module 10 involved in these KEGG pathways were the genes encoding guanine nucleotide-binding protein Gi, α2 subunit (GNAI2), p21-activated kinase 1 (PAK1), and β-actin (ACTB) (Table 4, Figure 5). ACTB is an actin of the cytoskeleton that provides a structural support to various cellular motions (Figure 5A, 5B, 5D, 5E). PAK1 can induce the reassembly of actin-based cytoskeleton to promote cellular movement (Figure 5A–5C, 5E). Giα2 plays a pivotal role in the process of chemokine-induced cellular migration (Figure 5C, 5D). Contrary to the microarray, the real-time qRT-PCR analysis revealed that *ACTB* mRNA abundance was up-regulated and *miR-661* was down-regulated in the DS group, but the differences were not statistically significant. In addition, contrary to what we expected, the mRNAs encoding GNAI2 and PAK1 were down-regulated and the miR-221 was up-regulated in the DS group. Both *GNAI2* and *PAK1* are target genes of miR-221. The results implicated that in patients with DS, infiltration capacity of APL cells in peripheral blood was reduced.

The above data analysis implicated that proliferation, differentiation, apoptosis and infiltration capacity of APL cells played important roles in DS pathogenesis. It has been generally accepted that APL-cell differentiation are a prerequisite for DS occurrence and with the use of differentiation-inducing agent, the infiltration capacity of partially differentiated APL cells is significantly enhanced. Furthermore, studies [14, 15] found that there is a significant positive correlation between DS occurrence and the peak WBC count in peripheral blood, implicated that the proliferation of APL cell during differentiation therapy was a promoting factor for DS pathogenesis. Our results definitely confirmed that proliferation, differentiation, and infiltration capacity of APL cells were associated with DS occurrence. In addition, apoptosis of APL cells was also found to be related to DS occurrence. In fact, cellar proliferation, differentiation, and apoptosis are inherently inseparable three processes.

Although our data revealed that proliferation, differentiation, apoptosis and infiltration capacity of APL cells played important roles in the DS pathogenesis, the actual changes in these biological processes in APL cells could not be figured out because for some biological process, the changes (enhance or reduce) inferred from the alterations in the expression of relevant genes were not always consistent. For example, regarding the cellular proliferation in model 1 (Figure 3A), the up-regulation of *E2F1* and *MYC* mRNAs and the down-regulation of *PKMYT1* mRNA seemed to indicate an enhanced proliferation of peripheral blood APL cells, whereas the concurrent down-regulation of *MCM4* mRNA seemed to predict suppression of cellular proliferation. Similarly, the opposite conclusions were also drawn from the data of modules 4 and 10. But why was it like this?

Our explanation is as follows. The entire process from the mRNA expression to the execution and control of various biological activities by proteins is incredibly complex. Although mRNA expression, protein expression, and protein activity are closely connected, they are not simply linearly correlated due to various translational and post-translational regulatory events. In addition, one kind of protein often has multiple, sometimes opposite functions and participates in multiple biological processes, and conversely, the completion of any biological process requires the coordination of a number of biological molecules, resulting in the formation of complex protein interaction networks. Therefore, any biological phenomenon is a combined action of a variety of proteins or biological molecules and their interaction, as well as the balance among various biological processes, rather than the expression of only one or few mRNAs or proteins. Systems biology emphasizes the systemic investigation of the mechanisms of disease occurrence and progression from a comprehensive viewpoint, is just based on these principles. However, it is worth noting that the above viewpoint does not mean that research on the involvement of a single gene or protein in a particular biological activity is meaningless. In fact, every experimental result is equally important because the result must imply certain mechanisms, although, that could currently be unknown.

Based on our results, further studies should focus on the difference of following biological processes of peripheral blood cells from APL patients between DS and non-DS groups: cellular proliferation, differentiation, or apoptosis, and the capability of cellular adhesion, migration, or responsiveness to chemokines. In fact it has long been believed that differentiation and infiltration capacity of APL cells were critical factors for DS occurrence. Then, the proteins that play important roles in the above biological processes of APL cells should be studied. The modular genes that repeatedly appeared in multiple KEGG pathways, including *E2F1*, *MYC*, *VEGFA*, *PAK1*, and *GNAI2*, and the modular miRNAs are all worthy of further research. Their roles in DS occurrence and progression have not been reported. Furthermore, in addition to the molecules involved in the enriched KEGG pathways, other genes in the modules are also worth attention. Because genes in the same module might have the same regulatory mechanisms or similar biological functions, modular functional information could be used to predict the function of an intramodular gene that is not involved in the enriched KEGG pathways, including the genes with unknown functions. This is another advantage of modularization analysis.

Compared with previous studies related to DS pathogenesis, the present study has the following virtues: First, the present research involves human subjects. To our knowledge, most related studies are *in vitro* cellular studies [15–20], only two studies have involved animal experiments [21, 22], and few involve human subjects. Second, a contrast analysis between patients in DS and non-DS groups was used in our study, whereas all previous studies on DS pathogenesis focused on the comparative analysis of APL cells before and after (or with and without) the ATRA or ATO treatment [15–22]. Third, the study subjects were selected strictly. The typicality of patients is essential to the reliability of the experimental results, in particular for DS-related studies. No definitive diagnostic criteria of DS are currently available, and due to the lack of specificity of the clinical manifestations, this complication is difficult to distinguish from concurrent bacteremia, sepsis, pneumonia, and pulmonary hemorrhage. In addition, there is no specific auxiliary examination approach for DS. Therefore, DS diagnosis is still difficult [23]. In this study, we spent 1.5 years in accumulating typical patients. Fourth, a system biology method was adopted, which starts studies from a more overall level. We also acknowledge limitations to this work. First, the predicted target genes of differentially expressed miRNAs were not experimentally verified before demonstrating regulatory network. Second, we did not use independent samples to validate the microarray results. As described above and in Materials and Methods, it is really hard to get enough samples that cut the mustard.

In this study, by integrative analysis of miRNA and mRNA expression profiles, we explore the pathogenesis of ATO treatment-associated DS in APL patients. The results could further our understanding of the mechanisms underlying DS occurrence and progression from a more comprehensive viewpoint, and could provide new cues for further research.

## MATERIALS AND METHODS

### Patient samples

Peripheral blood samples were obtained every day from all the patients who were diagnosed with APL in the First Affiliated Hospital of Harbin Medical University from September 2010 to April 2012 as soon as they were admitted to the hospital and informed consent was obtained. All patients received ATO for remission induction at the dose of 0.16 mg/kg/d, maximum 10 mg per day. The diagnosis of APL was established by the presence of the t(15;17) chromosomal abnormality or promyelocytic leukemia–retinoic acid receptor (PML-RARA) rearrangements. The mononuclear cells were isolated from fresh peripheral blood, lysed in Trizol Reagent (Invitrogen) and stored at −80°C until further use.

Based on whether DS had occurred during the treatment period, all patients who achieved hematological complete remission (HCR) after ATO induction treatment were grouped into two groups: one group with the complication of DS (DS group) and the other without DS (non-DS group). Because no definitive diagnostic standards for DS are currently available, we applied highly strict inclusion and exclusion criteria in the selection of patients for both DS and non-DS groups to ensure the reliability of the experimental results. In the DS group, patients had all of the following clinical or radiological presentations: dyspnea, weight gain greater than 5 kg due to edema, interstitial pulmonary infiltrates, and pleural and/or pericardial effusions, with or without fever, hypotension, and acute renal failure. Patients with any other medical problems, such as pulmonary hemorrhage, pneumonia, congestive heart failure, and renal failure, were excluded from this study. For the non-DS group, patients must have had none of the above presentations and weight gain no more than 2 kg.

Next, the blood samples collected from each patient who met the inclusion criteria were screened. Specifically, for the patients without the complication of DS, the blood samples that had highest peripheral blood WBC count were selected. For the patients complicated with DS, the blood samples collected at the day of DS diagnosis were used.

Then, the selected blood samples were further screened. Instead of all mononuclear cells in the peripheral blood, our experiments focused on differentiated or partially differentiated APL cells. To ensure a high purity of APL cells in the blood sample, only blood samples containing APL cells more than 95% of total mononuclear cells were selected in this study. At last, twelve blood samples, including six for the DS group and six for the non-DS group, were selected for study (Table S1).

The study protocol was approved by the Clinical Medical Ethics Committee of the First Affiliated Hospital of Harbin Medical University and all experiments were carried out in accordance with the approved guidelines.

### Microarray experiments

Total RNA was extracted from the mononuclear cells using Trizol reagent (Invitrogen).

For the gene expression profiling, the Illumina Human HT-12 v4 Expression BeadChip was used. It interrogates ≥47,000 target probes for genes obtained from the National Center for Biotechnology Information Reference Sequence RefSeq Release 38 and other sources. Total RNA was purified using Qiagen RNeasy Mini Kit (Qiagen) and 500 nanograms of total RNA were subjected to reverse transcription, second-strand cDNA synthesis, and in vitro transcription using Illumina TotalPrep RNA Amplification Kit (Ambion, USA). Biotin was incorporated into amplified RNA through the use of biotin-modified dUTP. Amplified RNAs were hybridized on the BeadChips according to the manufacturer's instructions. Then, chips were washed and stained with Cy3-streptavidin.

For the miRNA expression profiling, the Illumina Human v2 microRNA Expression BeadChip was used. It targets 1,146 human miRNAs. Total RNA inclusive of miRNA fraction was isolated using miRNeasy Mini Kit (Qiagen) and 200 nanograms of total RNA was polyadenylated and used as template to synthesize first-strand cDNA. Then, miRNA expression profiling was performed according to the Illumina protocols.

Both types of chips were scanned on an Illumina BeadArray Reader, and the chip intensity data were imported into Genome StudioTM Gene Expression Module v1.0 to perform quality control checks and data analysis. Raw data were normalized by the Quantile algorithm.

### Determination of the regulatory pairs of differentially expressed miRNAs/differentially expressed genes

The first step was to predict the targets of differentially expressed miRNA. We applied seven miRNA target prediction software programs (PicTar, RNAhybrid, DIANA-microT, RNA22, miRBase Targets, miRanda, and TargetScan) to predict the target genes of the detected differentially expressed miRNAs. To improve the reliability of the miRNA target prediction, only the target genes predicted from at least three programs were considered plausible and used for the subsequent analysis.

Next, the overlapping sets between the computationally predicted target genes of high-expressing miRNAs and the low-expressing genes in the DS group, as well as the overlapping sets between the predicted targets of low-expressing miRNAs and the high-expressing genes, were identified. As a result, the regulatory pairs of differentially expressed miRNAs/differentially expressed genes were identified.

### Determination of regulatory pairs of differentially expressed transcription factors/differentially expressed miRNAs

Because miRNA expression is regulated by transcription factors, and the mRNA expression information of transcription factors is also included in the mRNA expression profile chip array, we identified the regulatory pairs of differentially expressed transcription factors/differentially expressed miRNAs.

First, we searched the existing literature for the transcription factors regulating the expression of our differentially expressed miRNAs. A PubMed search with the keywords differentially expressed “microRNA” or “miRNA” and “transcription factor” returned articles that we manually searched to identify transcription factor/miRNA regulatory pairs here.

Second, we obtained the regulatory pairs of differentially expressed transcription factors/differentially expressed miRNAs based on the overlap between the transcription factors obtained from the above method and differentially expressed genes detected by the mRNA expression profile chip array.

### Construction of DS-related gene-regulatory network map

A DS-related gene-regulatory network was constructed based on the combination of the above-obtained regulatory pairs of differentially expressed transcription factors/differentially expressed miRNAs and the regulatory pairs of differentially expressed miRNAs/differentially expressed genes. In this network map, a node denoted a miRNA or gene (including a gene encoding a transcription factor), and a directed edge between the nodes denoted a regulatory relationship.

### Modularization of DS-related gene-regulatory network

Using the EAGLE algorithm in Cluster Viz-Cytoscape (http://code.google.com/p/clusterviz-cytoscape/), the entire network was subdivided into modules.

### Functional annotations of module by KEGG pathway enrichment analysis

Enrichment analysis was performed to find KEGG pathways that were significantly enriched in the gene sets of each module.

### Real-time qRT-PCR

For the quantification of mRNAs, total RNA was purified using RNeasy Mini Kit (Qiagen) and reverse transcribed to cDNA using QuantiTect Reverse Transcription Kit (Qiagen). Equal amounts of cDNA were taken for a subsequent real-time qRT-PCR using QuantiTect SYBR Green PCR kit (Qiagen). The relative quantity of mRNA was determined by the comparative threshold cycle method using glyceraldehyde-3-phosphate dehydrogenase (GAPDH) expression for normalization. All the primers used were listed in Table S2.

For miRNA analysis, total RNA inclusive of the small RNA fraction was purified using miRNeasy spin columns (Qiagen) and reverse transcribed using TaqMan® MicroRNA Reverse Transcription Kit (Applied Biosystems) according to the manufacturer's protocol. The TaqMan® MicroRNA Assays (Applied Biosystems) used for miRNA quantification were listed in Table S3. Expression levels of RNU6B were used for normalization.

The real-time qRT-PCR was run on a 7900HT Fast Real-Time PCR System (Applied Biosystems) and the data were analyzed using the 2^−ΔΔCt^ method.

### Statistical analysis

Student's *t*-test was used for analysis of difference in mRNA or miRNA expression between two groups. In microarray data analysis, differentially expressed mRNAs or miRNAs were selected using the criteria that the *P* values < 0.05 and fold change > 2 or < 0.5. KEGG pathway enrichment analysis was performed using cumulative hypergeometric distribution algorithm. *P* values < 0.05 were considered statistically significant.

## SUPPLEMENTARY MATERIALS TABLES


