# Supplementary material for: Integrating microRNA and mRNA expression profiles of acute promyelocytic leukemia cells to explore the occurrence mechanisms of differentiation syndrome
Source: Oncotarget. 2016 Sep 13;7(45):73509–24. doi: 10.18632/oncotarget.11989 (PMC5341995; doi:10.18632/oncotarget.11989)
Supplement: Supplementary file 1 [file oncotarget-07-73509-s001.pdf]

Integrating microRNA and mRNA expression profiles of acute promyelocytic leukemia cells to explore the occurrence mechanisms of differentiation syndrome

SUPPLEMENTARY TABLES

Supplementary Table S1: Clinical features of the 12 APL patients at admission or blood sampling

Items	Non-DS group (N=6)<br>Median (range) or number of patients	DS group (N=6)<br>Median (range) or number of patients
Age, years	29 (15-51)	41 (30-79)
Gender, male/female	3/3	2/4
Body weight at hospital admission (kg)	65 (55-90)	60.5 (38-70)
WBC count at hospital admission ($\times 10^9$/L)	1.7 (0.7-7.8)	14.8 (1.5-148)
<4	4	2
4-10	2	0
>10	0	4
Platelet count at hospital admission ($\times 10^9$/L)	27.1 (10.5-71)	19 (9-60)
≤40	5	5
>40	1	1
Number of patients with bleeding and/or DIC as evidenced by laboratory examinations	5	6
ECOG score of general physical condition at hospital admission (0/1/2/3/4)	0/3/2/1/0	0/2/2/1/1
FAB type (typical/atypical)	5/1	6/0
WBC count at the time of blood sampling ($\times 10^9$/L)	22.3 (9.7-38.5)	36.8 (9.3-99)
Duration of ATO treatment at the time of blood sampling (days)	13 (10-17)	10 (4-17)
Number of patients treated with a reduced dose of ATO or whose ATO treatment was discontinued	3	3
Number of patients receiving treatment for leukocytosis (hydroxycarbamide/low-dose DA regimen)	4/0	4/2
Number of patients receiving hormone therapy	0	3

Abbreviations: APL, acute promyelocytic leukemia; DS, differentiation syndrome; WBC, white blood cell; DIC, disseminated intravascular coagulation; ECOG, Eastern Cooperative Oncology Group; FAB, French-American-British; ATO, arsenic trioxide; DA, daunorubicin and cytarabine.

Supplementary Table S2: The PCR primers used in this study

No.	Genes	The forward primers	The reverse primers	Product size
1	E2F1	5'-GAGGAGACCGTAGGTGGGAT-3'	5'-GCTGAGTAGAGACTGGCTGG-3'	159
2	c-MYC	5'-GGGCTTTATCTAACTCGCTGTA-3'	5'-GCTATGGGCAAAGTTTCGTG-3'	222
3	VEGF-A	5'-CACACCCACCCACATACATACATT-3'	5'-TTCCAACTCAAGTCCACAGCAGTC-3'	170
4	CRKL	5'-AAGAACAGTGGTGGAGTGCC-3'	5'-GTGGTCTGAGGTTGAGCGTAT-3'	169
5	ACTB	5'-CTTAGTTGCGTTACACCCTTTCTTG-3'	5'-CTGTCACCTTCACCGTTCCAGTTT-3'	156
6	PAK1	5'-TTTCTGAACCGCTGTCTCG-3'	5'-TGTTCTTTGTTGCCTCCTTA-3'	191
7	GNAI2	5'-GCTGCCTACTACCTGAACGA-3'	5'-GACCACCCACATCAAACATC-3'	157
8	GAPDH	5'-GAAGGTCGGAGTCAACGGAT-3'	5'-CCTGGAAGATGGTGATGGGAT-3'	224

Supplementary Table S3: The TaqMan® MicroRNA Assays used in this study

No.	miRNA/mRNA	Assay ID
1	miR-15b	000390
2	miR-17	002308
3	miR-18a	002422
4	miR-93	001090
5	miR-106a	002169
6	miR-221	000524
7	miR-661	001606
8	miR-10a	000387
9	miR-133a	002246
10	miR-137	001129
11	miR-150	000473
12	miR-152	000475
13	miR-181d	001099
14	miR-202*	002362
15	miR-204	000508
16	miR-210	000512
17	miR-217	002337
18	miR-302c*	000534
19	miR-30c	000419
20	miR-30d	000420
21	miR-421	002700
22	miR-425	001516
23	miR-431	001979
24	miR-600	001556
25	RNU6B (control)	001093
